# Incidence risk of hepatobiliary malignant neoplasms in the cohort of workers chronically exposed to ionizing radiation

**DOI:** 10.1038/s41598-024-63503-z

**Published:** 2024-07-30

**Authors:** Galina Zhuntova, Maria Bannikova, Tamara Azizova

**Affiliations:** Clinical Department, Southern Urals Biophysics Institute Affiliated to the Federal Medical Biological Agency, Ozyorsk, Russian Federation 456780

**Keywords:** Hepatobiliary malignancies, Liver angiosarcoma, Hepatocellular carcinoma, Intrahepatic cholangiocarcinoma, Occupational radiation exposure, Internal alpha particle exposure, External gamma-ray exposure, Mayak workers cohort, Cancer, Pathogenesis, Risk factors

## Abstract

The increased risk of liver malignancies was found in workers of the first Russian nuclear production facility, Mayak Production Association, who had been chronically exposed to gamma rays externally and to alpha particles internally due to plutonium inhalation. In the present study, we updated the radiogenic risk estimates of the hepatobiliary malignancies using the extended follow-up period (1948–2018) of the Mayak worker cohort and the improved «Mayak worker dosimetry system–2013». The cohort comprised 22,377 workers hired at the Mayak PA between 1948 and 1982. The analysis considered 62 liver malignancies (32 hepatocellular carcinomas, 13 intrahepatic cholangiocarcinomas, 16 angiosarcomas, and 1 anaplastic cancer) and 33 gallbladder adenocarcinomas. The analysis proved the positive significant association of the liver malignancy risk (the total of histological types, hepatocellular carcinoma) with the liver absorbed alpha dose from internal exposure. The excess relative risk per Gy (95% confidence interval) of alpha dose (the linear model) was 7.56 (3.44; 17.63) for the total of histological types and 3.85 (0.95; 13.30) for hepatocellular carcinoma. Indications of non-linearity were observed in the dose–response for internal exposure to alpha radiation. No impact of external gamma-ray exposure on the liver malignancy incidence was found. In the study cohort, the number of angiosarcomas among various types of liver malignancies was very high (25.8%), and most of these tumors (73.3%) were registered in individuals internally exposed to alpha radiation at doses ranging between 6.0 and 21.0 Gy. No association with chronic occupational radiation exposure was observed for the incidence of gallbladder malignancies.

## Introduction

Liver carcinoma is one of the main causes of death from malignant neoplasms (MNs)^1^. The most common histological types of liver MNs are hepatocellular carcinoma (HCC) and cholangiocarcinoma of intrahepatic bile ducts (ICC); by comparison, liver angiosarcoma (AS) is very rare^2–4^.

Lifestyle (excessive alcohol consumption, smoking, nonalcoholic fatty liver disease due to unbalanced nutrition and low physical activity, and others), some chemicals (for example, aflatoxin) increase the risk of HBMNs development^5–8^. Infection with hepatitis B (HBV) and C (HCV) viruses plays a key role in HCC development^2,9^. Chronic inflammation of intrahepatic bile ducts, including that due to parasitic infections and gallstone disease, also contributes to the risk of ICC^3^. Exposure to ionizing radiation and vinyl chloride increases the risk of AS^4,8,10^.

A-bomb survivors from Hiroshima and Nagasaki acutely exposed to gamma-neutron radiation demonstrated an increased risk of HBMNs^11,12^. The extremely high risk of liver MNs was reported as a long-term side effect occurring after angiography with Thorotrast, the solution containing alpha-active thorium^10^. Workers of the first Russian nuclear production facility Mayak Production Association (PA) demonstrated an increased risk of incidence of and mortality from liver, lung and bone cancers which was associated with the alpha-particle dose from internal exposure to incorporated plutonium^13–15^. Meanwhile, other studies of occupationally exposed nuclear workers did not observe an association of the HBM risk with radiation exposure^16^.

The previous dose–response analysis performed for the Mayak worker cohort considered a small number of liver cancer cases^14,15^, and incidence risks for malignancies of the gall bladder were not estimated. At present, the cohort follow-up has been markedly extended, the Mayak worker dosimetry system (MWDS) has been improved, and data on non-radiation risk factors have been expanded; these updates served as the basis for the continuation of the study.

The objective of the present study was to update estimates of the radiogenic incidence risk of HBM due to chronic occupational external and internal exposure to gamma rays and alpha particles, respectively, considering the impact of non-radiation risk factors.

## Methods

### The study cohort and the follow-up period

Mayak Production Association (PA) is located near the city of Ozyorsk in the Chelyabinsk region (Russia); it started its operation in 1948. Since the early years of the facility operation, the health status of Mayak workers has been carefully monitored in accordance with the specifically developed protocol^17^. Data about Mayak workers collected throughout many years and continuously updated (demographic and medical information, lifestyle factors and radiation doses from occupational exposure) are stored as a medical and dosimetry database ‘Clinic’^17^ which is used to perform epidemiological studies.

This was a retrospective analysis of the cohort of workers hired at the main facilities of the Mayak PA (reactors, radiochemical plant, plutonium production facilities) from 1948 to 1982 (22,377 individuals; 25.4% were females). The follow-up period of a cohort member started on the date of hire at the main facility and ended on 31.12.2018 (date of HBMN diagnosis, date of death or date of the latest record in medical documentation for those who had deceased earlier or who had dropped out of the follow-up due to leaving the city of Ozyorsk for another place of residence).

### Dosimetry

Monitoring of external gamma exposure started immediately after the start-up of the Mayak operation and it was carried out using individual film badges. Workers employed at the radiochemical and plutonium production plants (76.0% of the cohort members) could have been internally exposed to alpha particles from incorporated plutonium in addition to external gamma exposure. Monitoring of internal alpha exposure started in the 1960s, and as a routine practice, it was implemented gradually. Alpha doses from internal exposure were estimated from urine measurements (24-h sample) of plutonium-239 activity in the body^18^.

To enable the estimation of organ- and tissue-absorbed gamma and alpha doses from external and internal exposures, respectively, the Mayak worker dosimetry system (MWDS) was developed. Over the years, the dosimetry system for occupational radiation exposure of Mayak workers has been elaborated and improved and individual dose estimates have been updated. To perform the analyses, we used MWDS-2013^18^ estimates of liver-absorbed gamma-ray and alpha-particle doses from external and internal (due to the incorporation of plutonium) exposures, respectively. Dose calculation techniques and specific features of the dosimetry monitoring organized for Mayak workers were reported earlier^18^.

### Statistical analysis

Statistical methods used in the present study were similar to those used earlier. The present analysis was restricted to the period of residency in the city of Ozyorsk since information about diseases, results of annual health check-ups and non-radiation factors was not available after a cohort member had moved from the city. We excluded from the analysis dataset 43 workers who had experienced acute gamma-neutron exposure at high dose rates resulting in acute radiation sickness, and 684 workers with missing medical records. So, the analysis of HBMN incidence risk due to external gamma exposure considered 21,650 workers. The analysis of the HBMN incidence risk in relation to internal alpha exposure considered only 7988 workers monitored for plutonium intakes.

The analysis of the HBMN incidence risk in relation to the occupational radiation dose was carried out using the Poisson regression, and the risk estimates were adjusted (via stratification) for the following non-radiation factors: sex, attained age (< 20, 20–25,…80–85, > 85), smoking status (never smoker, former smoker, smoker, unknown), alcohol consumption (seldom drinking, moderate drinking, abusive drinking, unknown), period of hire at the Mayak PA (1948–1960, 1961–1982), chronic liver and gallbladder diseases, gallstone disease and viral hepatitis in the anamnesis. Supplementary Table S2 summarizes tabulated data used in the present analysis.

Information about the smoking habit and alcohol consumption was collected and regularly updated at interviews during routine health checkups of workers^17^. Data on alcohol consumption (seldom or moderate drinking) were based on the workers’ subjective self-assessments. Those workers whose medical records had information relevant to abusive alcohol drinking or included inebriety and/or chronic alcoholism among other diagnoses were assigned to the corresponding group of alcohol consumption.

The analysis considered information about chronic diseases of the hepatobiliary tract if the relevant disease had been diagnosed at least 2 years before HBMN had been found (when the follow-up ended), to exclude a potential non-diagnosed neoplastic process that at the early stage could have indications similar to diseases listed above.

Occupational radiation doses were lagged for 10 years; dose uncertainties were not taken into account while analyzing the radiogenic risk. Workers for whom alpha doses from internal exposure were not available were assigned to the category “unknown alpha dose” rather than excluded from the analysis of the dose–response for gamma exposure.

The calculations were run with the AMFIT module of the EPICURE software^19^. The association of the HBMN incidence risk (λ) with the occupational radiation dose (D) was assessed using the linear model:

$$\uplambda = {\uplambda }_{0}\cdot \left(1 + {\upbeta }_{1}\text{D}\right),$$

where λ_0_ is the background risk, β_1_ is the excess relative risk per Gy (ERR/Gy), and D is the gamma or alpha dose. The background risk λ_0_ demonstrates the impact of non-radiation factors listed above and includes an adjustment for internal alpha dose while analyzing the risk due to external gamma-ray exposure and vice versa. 95% confidence intervals of ERR/Gy estimates were calculated with the maximum likelihood technique.

We tested the sensitivity of ERR/Gy estimates to changes in the set of factors considered in the model of the background risk, and compared the results between males and females.

Associations of the HBMN incidence risk with the internal alpha dose were also assessed using the linear-quadratic and the quadratic models:

$$\uplambda = {\uplambda }_{0}(\text{s},\text{ aa}, {\text{d}}_{\upgamma }) \cdot (1 + {\upbeta }_{1}{\text{D}}_{{\upalpha }} + {\upbeta }_{2}{\text{D}}_{{\upalpha }}^{2}),$$

$$\uplambda = {\uplambda }_{0}(\text{s},\text{ aa}, {\text{d}}_{\upgamma }) \cdot (1 + {\upbeta }_{2}{\text{D}}_{{\upalpha }}^{2}),$$

where λ_0_ is the background risk, β_1_ is the ERR/Gy and β_2_ is the ERR/Gy^2^, D_α_ is the alpha-particle dose. The background risk estimator λ_0_ includes adjustments only for sex (s), attained age (aa) and external gamma dose (d_γ_).

To compare the quality of the fit with various models, we used the likelihood function logarithm difference for nested models and the Akaike criteria for non-nested models^20^. The level of p < 0.05 indicated statistical significance.

Qualitative variables were presented as frequency; the quantitative variables were presented as mean ± standard deviation (SD), median, 25 and 75 quartiles (25–75%).

## Results

Table 1 and Supplementary Table S1 summarize the characteristics of the study cohort. As of 31.12.2018, complete information about past diseases was available for 97.3% of the cohort members, and the average age of workers alive on 31.12.2018 was 70.1 years while the average age of workers who had deceased earlier was 64.6 years (Table 1). Data about smoking and alcohol consumption were collected for more than 90% of the cohort members. (Table S1).

**Table 1:** Main characteristics of the study cohort.

Characteristics	Males	Females	Total
Number of workers included in the cohort	16,688	5689	22,377
Number of migrants^a^ (%)	7238 (43.4%)	2032 (35.7%)	9270 (41.4%)
Number of workers with available vital status as of 31.12.2018 (%)	15,887 (95.2%)	5450 (95.8%)	21,337 (95.4%)
Deceased	11,017 (69.3%)	3345 (61.4%)	14,362 (67.3%)
Alive	4870 (30.7%)	2105 (38.6%)	6975 (32.7%)
Number of workers with available data on diseases (%)	16,277 (97.5%)	5492 (96.5%)	21,769 (97.3%)
Average age at hire (SD), years	24.1 (7.1)	27.3 (8.0)	24.9 (7.5)
Average age at death for those who passed away (SD), years	62.3 (13.7)	72.1 (12.6)	64.6 (14.1)
Average age of alive workers as of 31.12.2018 (SD), years	67.6 (9.1)	75.8 (8.6)	70.1 (9.4)
Average age of migrants^a^ at the time of moving away from the city of Ozyorsk (SD), years	31.0 (10.0)	33.9 (11.3)	31.6 (10.3)
Average cumulative liver absorbed gamma dose from external exposure (SD), Gy^b^	0.45 (0.65)	0.37 (0.56)	0.43 (0.63)
Number of plutonium monitored workers (%)	5578 (33.4%)	2432 (42.7%)	8010 (35.8%)
Average cumulative liver absorbed alpha dose from internal exposure (SD), Gy^c^	0.18 (0.65)	0.40 (1.92)	0.25 (1.19)

^a^Denotes individuals who left Ozyorsk for another place of residence, SD denotes standard deviation.

^b^For all workers.

^c^For plutonium monitored workers.

Individual gamma-ray doses from external exposure were known for all workers of the study cohort, individual alpha doses were estimated only for 35.8% of the cohort members (Table S1). The range of radiation doses received by Mayak workers in the course of occupational activities was wide (Table S1). Before 1960, personnel radiation doses were the highest. Mean liver-absorbed gamma-ray and alpha-particle doses accumulated by the study cohort workers were 0.43 and 0.25 Gy, respectively (Table 1).

As at 31.12.2018, 62 liver carcinomas (C22 code of ICD-10) and 33 MNs of the gallbladder (as well as MNs of other and unspecified part of biliary tract—C23 and C24 codes of ICD-10) (hereinafter, GBMNs) were registered in the study worker cohort. All HBMNs considered in the present study were morphologically verified. The registered liver MNs varied in their histological structure: 32 HCCs (51.6%), 13 ICCs (21.0%), 16 ASs 25.8%), and 1 anaplastic cancer (1.6%) were identified. All GBMNs registered among study cohort workers were adenocarcinomas.

Table 2 summarizes the characteristics of liver MNs by a histological type and GBMNs. Among workers with HCCs and ICCs, the percentage of males was the highest, and the percentages of smokers and individuals abusing alcohol were also the highest. Among workers with ASs, females accounted for the majority, and the percentages of smokers and those who had abused alcohol were small, while the percentage of individuals diagnosed with chronic liver and gallbladder diseases and gallstone disease was high compared to other histological types of liver MNs. The majority of the workers with HBMNs were hired at the Mayak PA between 1948 and 1960 when occupational ionizing radiation doses were the highest. Workers with HCCs and particularly with ASs were predominantly employed at the plutonium production plant, and more than a third of workers with ICCs and GBMNs were employed at reactor facilities (Table 2).

**Table 2:** Characteristics of hepatobiliary malignancies among workers of the study cohort.

Factor	Liver malignancy				Gall bladder malignancy
	Total^a^	HCC	ICC	AS	
Number of cases	62	32	13	16	33
Number of person—years	598,323	597,267	596,366	596,601	595,752
Sex					
Males	43 (69.4%)	26 (81.2%)	12 (92.3%)	5 (31.2%)	21(63.6%)
Females	19 (30.6%)	6 (18.8%)	1 (7.7%)	11 (68.8%)	12 (36.4%)
Age at diagnosis					
Mean (SD)	63.9 (10.8)	66.4 (9.6)	59.5 (10.8)	63.3 (12.3)	63.2 (11.8)
Median (25%-75%)	64 (57–71)	65.5 (62–72)	59 (55–64)	61 (53–74)	63 (54–71)
Smoking					
Never smoker	27 (43.5%)	10 (31.2%)	3 (23.0%)	13 (81.3%)	12 (36.4%)
Former smoker	13 (21.0%)	8 (25.0%)	4 (30.8%)	1 (6.3%)	10 (30.3%)
Smoker	22 (35.5%)	14 (43.8%)	6 (46.2%)	2 (12.4%)	11 (23.3%)
Alcohol consumption					
Seldom drinking	19 (30.6%)	5 (15.6%)	1 (7.7%)	12 (75.0%)	13 (39.4%)
Moderate drinking	25 (40.4%)	17 (53.1%)	5 (38.5%)	3 (18.8%)	12 (36.4%)
Abusive drinking	18 (29.0%)	10 (31.3%)	7 (53.8%)	1 (6.2%)	8 (24.2%)
Viral hepatitis					
No	60 (96.8%)	30 (93.8%)	13 (100%)	16 (100%)	32 (97.0%)
Yes	2 (3.2%)	2 (6.2%)	–	–	1 (3.0%)
Chronic liver diseases					
No	54 (87.1%)	28 (87.5%)	12 (92.3%)	13 (81.3%)	32 (97.0%)
Yes	8 (12.9%)	4 (12.5%)	1 (7.7%)	3 (18.7%)	1 (3.0%)
Gallstone disease					
No	51 (82.3%)	28 (87.5%)	10 (76.9%)	12 (75.0%)	27 (81.8%)
Yes	11 (17.7%)	4 (12.5%)	3 (23.1%)	4 (25.0%)	6 (18.2%)
Chronic gallbladder diseases					
No	30 (48.4%)	18 (56.2%)	8 (61.5%)	3 (18.8%)	17 (51.5%)
Yes	32 (51.6%)	14 (43.8%)	5 (38.5%)	13 (81.3%)	16 (48.5%)
Calendar period of hire					
1948–1960	54 (87.1%)	25 (78.1%)	13 (100%)	15 (93.8%)	26 (78.8%)
1961–1982	8 (12.9%)	7 (21.9%)	–	1 (6.3%)	7 (21.2%)
Facility type					
Reactors	12 (19.4%)	6 (18.8%)	5 (38.4%)	1 (6.2%)	11 (33.3%)
Radiochemical plant	22 (35.5%)	13 (40.6%)	4 (30.8%)	4 (25.0%)	14 (42.4%)
Plutonium production	28 (45.1%)	13 (40.6%)	4 (30.8%)	11 (68.8%)	8 (24.2%)

^a^The total includes one case of the anaplastic cancer.

Within the study worker cohort, 21 liver MNs (33.8%) and 4 GBMNs (12.2%) were identified in workers externally exposed to gamma rays at doses above 1.0 Gy (Table 3).

**Table 3:** The distribution of hepatobiliary malignancies among workers of the study cohort by the liver absorbed radiation dose.

Liver absorbed gamma dose from external exposure, Gy	Liver malignancy				Gallbladder malignancy
	Total^a^	HCC	CC	AS	
< 0.1	8 (12.9%)	6 (18.8%)	2 (15.4%)	0 (0%)	6 (18.2%)
0.1–0.5	17 (27.5%)	12 (37.4%)	3 (23.1%)	2 (12.5%)	8 (24.2%)
0.5–1.0	16 (25.8%)	6 (18.8%)	3 (23.1%)	7 (43.8%)	15 (45.4%)
1.0–2.0	18 (29.0%)	6 (18.8%)	5 (38.4%)	6 (37.5%)	2 (6.1%)
2.0–4.0	2 (3.2%)	2 (6.2%)	0 (0%)	0 (0%)	2 (6.1%)
> 4.00	1 (1.6%)	0 (0%)	0 (0%)	1 (6.2%)	0 (0%)
Total	62 (100%)	32 (100%)	13 (100%)	16 (100%)	33 (100%)
Mean (SD)	0.88 (0.82)	0.65 (0.71)	0.86 (0.57)	1.27 (1.06)	0.65 (0.54)
Median (25–75%)	0.68 (0.19–1.38)	0.42 (0.13–0.97)	0.93 (0.19–1.26)	0.95 (0.63–1.60)	0.62 (0.17–0.88)

Liver absorbed alpha dose from internal exposure^a^, Gy	Liver malignancy				Gallbladder malignancy
	Total^b^	HCC	CC	AS	
Not measured	13 (21.0%)	7 (21.9%)	5 (38.5%)	1 (6.3%)	14 (42.4%)
< 0.1	22 (35.5%)	15 (46.9%)	6 (46.1%)	1 (6.3%)	11 (33.3%)
0.1–0.5	6 (9.7%)	4 (12.5%)	1 (7.7%)	1 (6.3%)	7 (21.2%)
0.5–1.0	2 (3.2%)	1 (3.1%)	0 (0%)	0 (0%)	1 (3.1%)
1.0–2.0	2 (3.2%)	1 (3.1%)	1 (7.7%)	0 (0%)	0 (0%)
2.0–4.0	2 (3.2%)	0 (0%)	0 (0%)	2 (12.5%)	0 (0%)
4.0–6.0	1 (1.6%)	1 (3.1%)	0 (0%)	0 (0%)	0 (0%)
6.0–10.0	5 (8.1%)	1 (3.1%)	0 (0%)	4 (25.0%)	0 (0%)
> 10.0	9 (14.5%)	2 (6.3%)	0 (0%)	7 (43.7%)	0 (0%)
Total	62 (100%)	32 (100%)	13 (100%)	16 (100%)	33 (100%)
Mean (SD)^b^	4.24 (6.64)	2.09 (5.17)	0.17 (0.36)	10.2 (6.94)	0.23 (0.34)
Median (25–75%)	0.16 (0.02–7.39)	0.05 (0.02–5.17)	0.02 (0.01–0.13)	8.77 (3.62–16.14)	0.07 (0.03–0.25)

^a^Only for monitored workers.

^b^The total includes one case of anaplastic liver cancer.

We should note that estimates of alpha doses of internal exposure were unavailable for 27 workers with HBMNs. Among workers diagnosed with liver MNs and available alpha doses, 73.3% of ASs and 12.0% HCCs were identified in those internally exposed to alpha particles at liver absorbed doses 6.0–21.0 Gy. On the contrary, all ICCs and GBMNs (ACs) were registered in workers with a cumulative liver-absorbed alpha dose below 1.0–2.0 Gy (Table 3).

Tables 4 and 5 summarize estimates of excess relative risk of liver MN (the total of MNs of any histological types) and GBMNs per 1 Gy of external gamma-ray and internal alpha-particle exposure (ERR/Gy) which were calculated using linear models that included various sets of variables for baseline risk factors. Estimates of ERR/Gy of external gamma exposure were positive and statistically significant when the model included adjustments for sex, attained age, and all other considered non-radiation factors while analyzing the risk of liver MNs or adjustments for sex, attained age, viral hepatitis, chronic liver and gallstone diseases while analyzing the GBMN risk. (Table 4). After the adjustment for the alpha dose from internal exposure had been included in the model, the ERRs/Gy of external gamma exposure became statistically non-significant both for liver MN and GBMN incidence.

**Table 4:** The excess relative risk/Gy of the incidence of hepatobiliary malignancies by the gamma dose from external exposure (linear model).

Baseline risk	Excess relative risk /Gy (95% confidence interval), linear model	
	Liver malignancy	Gallbaldder malignancy
Sex, attained age	**3.18 (1.15; 9.06)**	**1.48 (0.05; 6.38)**
Sex, attained age, alcohol	**2.55 (0.87; 7.20)**	1.09 (− 0.08; 5.06)
Sex, attained age, smoking	**3.35 (1.21; 9.60)**	1.23 (− 0.04; 5.59)
Sex, attained age, viral hepatitis	**3.03 (1.07; 8.68)**	**1.48 (0.05; 6.39)**
Sex, attained age, chronic liver diseases	**3.12 (1.12; 8.86)**	**1.49 (0.05; 6.43)**
Sex, attained age, gallstone disease	**3.15 (1.13; 8.98)**	**1.38 (0.02; 6.04)**
Sex, attained age, chronic gallbladder disease	**2.89 (1.02; 8.21)**	1.32 (− 0.002; 5.80)
Sex, attained age, calendar period of hire	**1.21 (0.24; 4.10)**	0.48 (− 0.63; 4.37)
Sex, attained age, internal alpha dose	− 0.09 (− 0.28; 0.77)	0.77 (− 0.72; 5.07)
Sex, attained age, internal alpha dose, calendar period of hire, smoking, alcohol, viral hepatitis, chronic diseases of liver and gallbladder, gallstone disease	− 0.21 (n/a; 0.10)	− 0.21 (n/a; 1.56)

Significant values are in bold.

**Table 5:** The excess relative risk/Gy of the incidence of hepatobiliary malignancies by the alpha dose from internal exposure (linear model).

Baseline risk	Excess relative risk/Gy (95% confidence interval), linear model	
	Liver malignancy	Gallbaldder malignancy
Sex, attained age	**12.54 (6.10; 27.17)**	2.00 (− 2.15; 14.03)
Sex, attained age, alcohol	**11.34 (5.46; 24.79)**	0.56 (− 2.49; 8.32)
Sex, attained age, smoking	**13.17 (6.36; 28.98)**	1.67 (− 2.10; 12.56)
Sex, attained age, viral hepatitis	**12.37 (5.98; 26.9)**	2.36 (− 2.22; 15.64)
Sex, attained age, chronic liver diseases	**11.59 (5.61; 25.11)**	2.05 (− 2.19; 14.18)
Sex, attained age, gallstone disease	**11.94 (5.81; 25.84)**	1.98 (− 2.19; 14.18)
Sex, attained age, chronic gallbladder disease	**12.24 (5.95; 26.49)**	2.04 (− 2.20; 14.11)
Sex, attained age, calendar period of hire	**8.17 (3.91; 17.87)**	− 0.004 (n/a; 6.003)
Sex, attained age, external gamma dose	**7.56 (3.44; 17.63)**	− 0.04 (n/a; 5.22)
Sex, attained age, external gamma dose, calendar period of hire, smoking, alcohol, viral hepatitis, chronic diseases of liver and gallbladder gallstone disease	**7.44 (2.93; 20.63)**	− 0.04 (n/a; 4.25)
Risk modification by sex:		
Males	**3.91 (1.24; 11.15)**	− 0.06 (n/a; 13.96)
Females	26.62 (− 2.65; 627)	− 0.04 (n/a; 12.94)
*p* (test for heterogeneity)	**0.044**	> 0.5

Significant values are in bold.

The analysis performed with the linear model demonstrated the significant association of the liver MN with alpha dose from internal exposure (Table 5). Estimates of liver MN ERR/Gy of internal alpha exposure adjusted for sex, attained age, alcohol consumption, smoking and diseases were similar in magnitude. However, once the adjustment for the period of hire or the external gamma dose had been included, the ERR/Gy estimate decreased (by 35% and 40%, respectively) but remained statistically significant.

Significant differences in estimates of ERR/Gy of internal alpha exposure were observed between males and females (p = 0.044), but only in males the association of the incidence risk of liver MNs with the alpha dose was significant (Table 5).

The analysis did not demonstrate an effect of internal exposure to alpha particles on the GBMN incidence among the study cohort workers (Table 5).

**Figure 1:**
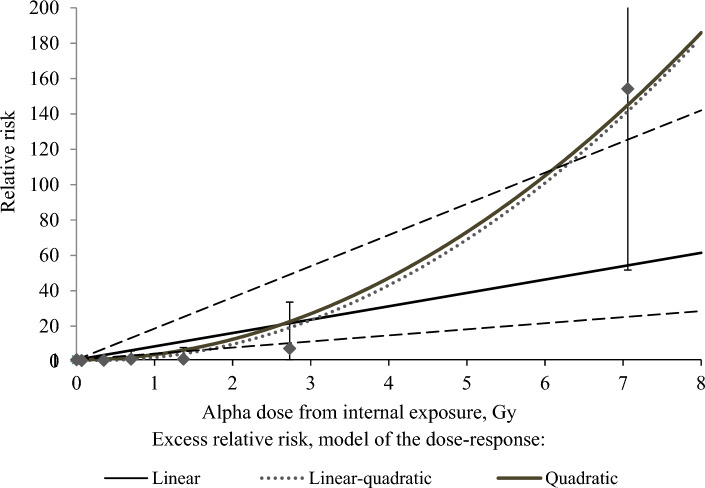
The risk of liver malignancy in relation to the alpha dose from internal exposure.

Table 6 summarizes the results of the incidence risk analysis for the total liver MNs and for certain histological types of tumors in relation to internal alpha-particle exposure provided with the linear and non-linear (quadratic and linear-quadratic) models. The analysis revealed a linear significant association with the alpha dose for the total liver MN and for the HCC incidence (Table 6). The estimate of ERR/Gy of internal alpha exposure (provided with the linear model) for HCCs was 2 times lower than for the total liver MNs. Non-linear models provided better data fits for the total liver MNs (all histological types), HCCs and ASs compared to the linear model. Some parameters of non-linear models were not statistically significant. For this reason, no adequate dose–response model could be determined for ASs. Significant quadratic risk associations with internal alpha dose (over the entire dose range) were found for the total liver MNs and for HCCs (Table 6). Figure 1 demonstrates the analysis results for the liver MN incidence risk association with the alpha dose from internal exposure (over the entire dose range). No significant liver MN incidence risk associations with the alpha dose were observed within dose intervals of 0–2 and 0–4 Gy (Table 6).

**Table 6:** The excess relative risk of the incidence of hepatobiliary malignancies by the alpha dose from internal exposure (comparison between linear and non-linear models).

Model	Liver absorbed alpha dose from internal exposure	Dose coefficients		Deviance	Comparison criteria
		β1	β2		
Liver malignancies (the total)					
Linear	Whole dose range	**7.56 (3.44; 17.63)**	–	683.32	–
	0–4 Gy	1.61 (− 0.31; 5.89)	–	541.28	–
	0–2 Gy	0.61 (− 1.02; 4.62)	–	515.15	–
Quadratic	Whole dose range	–	**2.89 (1.33; 6.32)**	653.99	**ΔAIC = 29.439**
	0–4 Gy	–	1.13 (− 0.10; 3.99)	539.84	ΔAIC = 1.441
	0–2 Gy	–	0.54 (− 1.35; 4.56)	515.27	ΔAIC = 0.119
Linear-quadratic	Whole dose range	− 1.99 (− 3.80; 0.95)	**3.06 (1.51; 6.17)**	651.78	p < 0.001
	0–4 Gy	− 0.46 (− 3.25; 4.70)	1.36 (− 0.80; 4.73)	539.77	p = 0.22
	0–2 Gy	1.28 (− 3.96; 6.53 )	− 0.74 (− 4.34; 2.86)	515.10	p > 0.5
Hepatocellular carcinoma					
Linear	Whole dose range	**3.85 (0.95; 13.30)**	–	373.45	–
Quadratic		–	**2.14 (0.54; 6.96)**	362.49	**ΔAIC = 10.964**
Linear-quadratic		− 1.89 (− 4.30; 2.30)	**2.49 (0.70; 7.08)**	361.18	p < 0.001
Cholangiocarcinoma					
Linear	Whole dose range	− 0.04 (na; 6.43)	–	142.03	*–*
Quadratic		–	− 0.002 (− 0.17; 0.16)	142.03	ΔAIC = 0.173
Linear-quadratic		− 0.01 (− 2.49; 2.47)	− 0.001 (− 0.29; 0.29)	142.03	p > 0.5
Angiosarcoma					
Linear	Whole dose range	1.68*10^7^ (na)	–	166.42	–
Quadratic		–	11.77 (− 9.45; 32.99)	148.03	**ΔAIC = 10.964**
Linear-quadratic		− 5.17 (− 7.81; − 2.53)	**6.68 (0.22; 13.15)**	144.91	p < 0.001

*p* denotes statistical significance of differences from the linear model (according to the maximum likelihood test); *ΔAIC* denotes the difference in Akaike criteria between the linear and quadratic models.

Significant values are in bold.

## Discussion

Earlier the cohort of Mayak workers was considered in a number of epidemiological studies which observed the increased risk of liver MNs associated with exposure to alpha particles. Gilbert et al.^13^ considered workers hired at the Mayak PA in 1948–1958. The analysis included 60 deaths from liver MNs reported before 1996, among them 24 deaths from HCCs, 8 deaths from ICCs, 10 deaths from ASs (with 8 deaths in females), 2 deaths from anaplastic cancer and 16 deaths from malignancies of the unknown histological type (deaths from cancer according to death certificates). The study reported the increased risk of mortality from liver cancer in workers with plutonium body activity above 7.4 kBq and the positive significant trend in the relative risk with plutonium body activity. Relative risk estimates were higher in females than in males, especially at the plutonium production facility.

Sokolnikov et al.^14^ investigated the mortality of Mayak PA workers hired in 1948–1972 and followed up until 2003. During this period, 75 deaths from liver MNs were registered, among those doses from incorporated plutonium were available for 26 workers. The analysis of the dataset limited to these workers found the linear association of the liver cancer mortality risk with the alpha dose from internal exposure: ERR/Gy = 2.6 (95% CI 0.7, 6.9) in males and ERR/Gy = 29 (95% CI 9.8, 95) in females (the differences were significant).

In their study, Labutina et al.^15^ considered workers hired at the Mayak PA in 1948–1982 and the follow-up period until 2004 and used estimates of occupational radiation doses provided by the MWDS-2008. The analysis included 52 liver cancers with tumor morphology available for 46 of them: 28 tumors were HCCs, 8 tumors were ICCs, and 10 tumors were ASs. The authors estimated radiogenic risks which were also adjusted for smoking and alcohol consumption. Those workers who had not been monitored for incorporated plutonium were divided into 6 categories by the inferred alpha dose from internal exposure based on the occupational history (surrogate dose categories). The linear model provided the following estimates of the total liver MN risks associated with the alpha dose from internal exposure: ERR/Gy = 6.1 (95% CI 2.0, 23.1) for males, ERR/Gy = 24.0 (95% CI 6.4, 141.2) for females, and ERR/Gy = 10.6 (95% CI 4.7, 30.0) for both sexes. The linear-quadratic model provided the best fit of the data on liver MN incidence risk with the radiation dose, however, the observed association was linear in the range of doses below 2.0 Gy. To fit the HCC incidence risk association with the alpha dose from internal exposure, the authors used the linear-quadratic model, but the linear coefficient in this model was non-significant. For ICC and AS, dose–response relationships were not analyzed.

In the present study, as compared to the study by Labutina et al.^15^, the follow-up of the Mayak worker cohort was extended for 14 years, and as a result, the number of cases included in the analysis increased (the numbers of ICCs and ASs increased by 1.6, and the number of HCCs increased by 1.2 times); only morphologically verified tumors were considered; updated radiation dose estimates provided by the improved MWDS-2013 were used; the list of non-radiation factors included in the model as adjustments was expanded (viral hepatitis, chronic diseases of the hepatobiliary tract); the risk due to internal alpha exposure was estimated for workers who had been monitored for plutonium intakes.

We should note that liver MN ERR/Gy of internal alpha exposure estimated with the linear model in the compared studies were similar in magnitude. However, in contrast to the results of earlier studies^14,15^, the ERR/Gy estimated in the present study for females was non-significant. Similar to the earlier findings^15^, here we observed the evidence of a non-linear association of the liver MN incidence risk with the alpha dose from internal exposure. The quadratic and linear-quadratic models provided the best fit of the dose–response (for total liver MNs and HCCs). It should be emphasized that ASs were predominantly registered in workers internally exposed to alpha particles at extremely high doses (the median of 8.77 Gy, 3.62–16.14 Gy—25–75%) in combination with considerable external gamma ray exposure (the median of 0.95 Gy, 0.63–1.60 Gy—25–75%).

The majority of the study cohort were workers exposed to mixed radiation types (externally to gamma rays and internally to alpha particles). The strongest impact on the radiogenic risk estimates was observed with an adjustment for another radiation type as compared to adjustments for non-radiation risk factors. When the estimates were not adjusted for alpha dose, ERRs/Gy of the external gamma exposure were significant. The magnitude of the ERR/Gy of internal alpha exposure estimated for the liver MN incidence reduced by 1.6 times once the adjustment for external gamma exposure had been included.

So, the differences in the radiogenic risk estimates for liver malignancies in Mayak workers between the studies discussed above can be due to the changes in occupational radiation dose estimates following the improvements of the MWDS. In addition, cohort follow-up periods and the numbers of liver MNs considered in the analyses were different. Gilbert et al.^13^ and Sokolnikov et al.^14^ reported the analyses of mortality from liver MNs using death certificates (which means that morphological verification of reported liver MNs was not carried out in 100% of cases). The analysis by Labutina et al.^15^ and the present analysis of the radiogenic risk considered morphologically verified incident cases of liver MNs. In the present study, the alpha dose–response analysis was limited to workers with available individually estimated alpha doses while Labutina et al.^15^ used surrogate alpha dose estimates for workers non-monitored for plutonium body intakes.

As for other nuclear worker cohorts, with plutonium production workers among them, no impact of occupational radiation exposure on HBMNs was observed^16,21–24^. As it was discussed repeatedly earlier, radiation doses accumulated by workers of these facilities were notably lower than those experienced by workers of the Mayak PA during the early period of the production start-up and development of technological processes^25^.

The long-term effects of Thorotrast were investigated in detail. Following the administration of this radiographic contrast agent containing the alpha-active thorium, patients demonstrated a considerable (tens-hundreds times) increase in the liver MN risk, mostly ICCs and ASs, and the risk of GBMNs was also elevated^10,26–28^. Differences in the percentage of histological types of liver MNs between Mayak workers and patients following the Thorotrast administration could be explained by peculiarities of micro-distribution of plutonium (which deposits in hepatocytes) and colloid particles of thorium (which deposits in bile ducts)^29,30^.

The Japanese atomic bomb survivors demonstrated almost proportional elevation of the risks of main histological types of liver MNs—HCCs and ICCs, which was because various liver structures had been exposed to the same levels of high-LET radiation^31^. Epidemiological studies of the Japanese atomic bomb survivors demonstrated the significant linear associations of liver cancer incidence and mortality with the gamma-neutron dose from acute exposure^11,12^, meanwhile for GBMNs, the association was observed only in mortality analyses^12^. The ERR/Gy magnitude was found to be sensitive to age, while smoking, alcohol consumption and body mass index did not have a tangible effect on the risk estimate (HBV and HCV were not considered)^11^. Some studies demonstrated the multiplicative effect of acute gamma-neutron exposure and HCV infection, however, in general, the data are controversial and require further investigations^32^.

The present retrospective study considered information about the viral hepatitis reported in medical records, however, the details with regards to the type of infection and carrier potential were not available for all workers. Among individuals diagnosed with the HBMNs, viral hepatitis was registered in two workers with HCC (in one of them, it was diagnosed 63 years before liver cancer and the virus type was unknown; in another worker, the hepatitis C carriage was registered 9 years before liver cancer diagnosis) and on one worker with GBMN (15 years before cancer diagnosis; the virus type was unknown).

It’s important to note that the study has some limitations: the dose–response analysis was based on a small number of liver MNs which were unevenly distributed over the wide range of alpha doses from internal exposure; incomplete data on internal alpha doses; unaccounted uncertainties of occupational dose estimates; lack of reliable information about workers who had been infected with hepatitis viruses.

The present study has a number of strengths which are the large size of the cohort, the long follow-up period, the availability of individually measured occupational radiation doses, the wide range of the doses, morphological verification of all HBMNs included in the analysis, and complete and high-quality information about registered diseases and about non-radiation factors.

## Conclusion

The present study confirmed the positive significant association of the liver MN incidence risk with the alpha dose from internal exposure to incorporated plutonium. The dose–response for total liver MNs and HCCs was best fitted with the quadratic model. ASs were mostly registered in workers internally exposed to alpha particles in the dose range of 6.0–21.0 Gy. The incidence risks of ICCs and GBMNs (ACs) were not associated with the internal alpha dose. No impact of chronic external gamma-ray exposure (taking into account internal alpha exposure) on the risk of HBMN incidence was observed for individuals occupationally exposed to ionizing radiation.

### Supplementary Information

Supplementary Tables.

## Data Availability

The dataset is the intellectual property of the Southern Urals Biophysics Institute (Ozyorsk, Chelyabinsk Region, 456780, Russia). For privacy reasons, it is not publicly available. These restrictions on data availability are imposed by Federal Act No. 323 of 21 November 2011 on the basics of health care for Russian citizens and Federal Act No. 152 of 27 July 2014 on personal data. Any access to the Mayak Worker Cohort must be approved by the institutional Ethics Review Board of the Southern Urals Biophysics Institute. To request the data used in the presented analyses, contact Drs. Tamara Azizova (the head of the clinical department of the Southern Urals Biophysics Institute) and Yuliya Tsareva (researcher of the Southern Urals Biophysics Institute, member of the institutional Ethics Review Board, tsareva@subi.su).
