# Supplementary material for: Incidence risk of hepatobiliary malignant neoplasms in the cohort of workers chronically exposed to ionizing radiation
Source: Sci Rep. 2024 Jul 30;14:17561. doi: 10.1038/s41598-024-63503-z (PMC11289462; doi:10.1038/s41598-024-63503-z)
Supplement: Supplementary file 1 — Supplementary Tables. [file 41598_2024_63503_MOESM1_ESM.docx]

**Incidence Risk of Hepatobiliary Malignant Neoplasms in the Cohort of Workers Chronically Exposed to Ionizing Radiation**

SUPPLEMENTARY MATERIAL

| Table S1. Characteristics of the study cohort | | | | | | |
| --- | --- | --- | --- | --- | --- | --- |
| Distribution of workers by smoking status and by sex | | | | | | |
| Smoking status | Males | | Females | | Both sexes | |
|  | Number | % | Number | % | Number | % |
| Never smoker | 3,562 | 21.3 | 5,089 | 89.5 | 8,651 | 38.7 |
| Former smoker | 3,012 | 18.1 | 132 | 2.3 | 3,144 | 14.1 |
| Smoker | 9,170 | 55.0 | 169 | 2.9 | 9,339 | 41.7 |
| Unknown | 944 | 5.6 | 299 | 5.3 | 1,243 | 5.5 |
| Total | 16,688 | 100.0 | 5,689 | 100.0 | 22,377 | 100.0 |
| Distribution of workers by alcohol consumption and by sex | | | | | | |
| Alcohol consumption | Males | | Females | | Both sexes | |
|  | Number | % | Number | % | Number | % |
| Seldom drinking (+not drinking ever) | 1,956 | 11.7 | 3,767 | 66.2 | 5,723 | 25.6 |
| Moderate drinking | 8,785 | 52.6 | 1,332 | 23.4 | 10,117 | 45.2 |
| Abusive drinking | 4,368 | 26.2 | 213 | 3.7 | 4,581 | 20.5 |
| Unknown | 1,579 | 9.5 | 377 | 6.7 | 1,956 | 8.7 |
| Total | 16,688 | 100.0 | 5,689 | 100.0 | 22,377 | 100.0 |
| Distribution of workers by age when they were hired at the facility and by sex | | | | | | |
| Age at hire, years | Males | | Females | | Both sexes | |
|  | Number | % | Number | % | Number | % |
| ≤ 20 | 6,642 | 39.8 | 1,162 | 20.4 | 7,804 | 34.9 |
| 21 – 30 | 7,491 | 44.8 | 2,857 | 50.2 | 10,348 | 46.2 |
| 31 – 40 | 1,795 | 10.8 | 1,207 | 21.2 | 3,002 | 13.4 |
| Older 40 | 760 | 4.6 | 463 | 8.2 | 1,223 | 5.5 |
| Total | 16,688 | 100.0 | 5,689 | 100.0 | 22,377 | 100.0 |
| Distribution of workers by the calendar period of hire and by sex | | | | | | |
| Period of hire, years | Males | | Females | | Both sexes | |
|  | Number | % | Number | % | Number | % |
| 1948 – 1960 | 10,352 | 62.0 | 3,810 | 67.0 | 12,297 | 55.0 |
| 1961 – 1982 | 6,336 | 38.0 | 1,879 | 33.0 | 10,080 | 45.0 |
| Total | 16,688 | 100.0 | 5,689 | 100.0 | 22,377 | 100.0 |
| Distribution of workers by age at the end of the follow-up and by sex | | | | | | |
| Age at the end of the follow-up, years | Males | | Females | | Both sexes | |
|  | Number | % | Number | % | Number | % |
| <50 | 2,688 | 16.1 | 405 | 7.1 | 3,093 | 13.8 |
| 50-59 | 3,191 | 19.1 | 439 | 7.7 | 3,630 | 16.2 |
| 60-69 | 5,449 | 32.7 | 1053 | 18.5 | 6,502 | 29.1 |
| Older 70 | 5,360 | 32.1 | 3792 | 66.7 | 9,152 | 40.9 |
| Total | 16,688 | 100.0 | 5689 | 100.0 | 22,377 | 100.0 |
| Distribution of workers by type of facility and by sex | | | | | | |
| Type of facility | Males | | Females | | Both sexes | |
|  | Number | % | Number | % | Number | % |
| Reactors | 4,194 | 25.1 | 1,170 | 20.5 | 5,364 | 24.0 |
| Radiochemical plant | 6,857 | 41.1 | 2,360 | 41.5 | 9,217 | 41.2 |
| Plutonium production | 5,637 | 33.8 | 2,159 | 38.0 | 7,796 | 34.8 |
| Total | 16,688 | 100.0 | 5,689 | 100.0 | 22,377 | 100.0 |
| Distribution of workers by liver absorbed gamma dose from external exposure and by sex | | | | | | |
| Liver absorbed gamma dose from external exposure, Gy | Males | | Females | | Both sexes | |
|  | Number | % | Number | % | Number | % |
| <0.1 | 6,045 | 36.2 | 2,628 | 46.2 | 8,673 | 38.8 |
| 0.1 – 0.5 | 6,025 | 36.1 | 1,684 | 29.6 | 7,709 | 34.5 |
| 0.5 – 1.0 | 2,122 | 12.7 | 676 | 11.9 | 2,798 | 12.4 |
| 1.0 – 2.0 | 1,828 | 11.0 | 558 | 9.8 | 2,386 | 10.6 |
| 2.0 – 4.0 | 633 | 3.8 | 138 | 2.4 | 771 | 3.5 |
| >4.00 | 35 | 0.2 | 5 | 0.1 | 40 | 0.2 |
| Total | 16,688 | 100.0 | 5,689 | 100.0 | 22,377 | 100.0 |
| Distribution of workers by liver absorbed alpha dose from internal exposure and by sex | | | | | | |
| Liver absorbed alpha dose from internal exposure, Gy | Males | | Females | | Both sexes | |
|  | Number | % | Number | % | Number | % |
| <0.1 | 4,082 | 73.2 | 1,683 | 69.2 | 5,765 | 72.0 |
| 0.1 – 0.5 | 1,081 | 19.4 | 521 | 21.4 | 1,602 | 20.0 |
| 0.5 – 1.0 | 206 | 3.6 | 95 | 3.9 | 301 | 3.8 |
| 1.0 – 2.0 | 121 | 2.2 | 52 | 2.1 | 173 | 2.2 |
| 2.0 – 4.0 | 2 | 0.1 | 24 | 1.0 | 26 | 0.3 |
| 4.0 – 6.0 | 62 | 1.1 | 27 | 1.1 | 89 | 1.1 |
| 6.0 – 10.0 | 12 | 0.2 | 16 | 0.7 | 28 | 0.3 |
| >10.0 | 12 | 0.2 | 14 | 0.6 | 26 | 0.3 |
| Total | 5,578 | 100.0 | 2,432 | 100.0 | 8,010 | 100.0 |

| Table S2. Variables included in the analysis | | | |
| --- | --- | --- | --- |
| Type | Variable | Description | |
| Fixed | Sex | 1: М | 2: F |
| Time dependent | Attained age | 1: <20  2: 20–25  3: 25–30  4: 30–35  5: 35–40  6: 40–45  7: 45–50  8: 50–55 | 9: 55–60  10: 60–65  11: 65–70  12: 70–75  13: 75–80  14: 80–85  15: ≥85 |
| Fixed | Period of hire | 1: 1948–1960 | 2: 1961–1982 |
| Fixed | Smoking status | 1 : never smoker  2: former smoker | 3: smoker  4: unknown |
| Fixed | Alcohol consumption | 1: never/seldom  2: moderate | 3: abusive  4: unknown |
| Fixed | Viral hepatitis | 1: no | 2: yes |
| Fixed | Chronic liver diseases | 1: no | 2: yes |
| Fixed | Gallstone disease | 1: no | 2: yes |
| Fixed | Chronic gallbladder diseases | 1: no | 2: yes |
| Fixed | Facility | 1: Reactors  2: Radiochemical plant | 3: Plutonium production |
| Time dependent | Liver absorbed gamma dose from external exposure (Gy), 10-year lag | 1: 0–0.1  2: 0.1–0.5  3: 0.5–1.0 | 4: 1.0–2.0  5: 2.0–4.0  6: ≥4.0 |
| Time dependent | Liver absorbed alpha dose from internal exposure (Gy), 10-year lag | 1: not measured  2: 0.00–0.1  3: 0.1–0.5  4: 0.5–1.0  5: 1.0–2.0 | 6: 2.0–4.0  7: 4.0 – 6.0  8: 6.0 – 10.0  9: ≥10.0 |
| Calculated | Attained age | Person-years weighted by the average attained age | |
|  | Liver absorbed gamma dose from external exposure (Gy), 10-year lag | Person-years weighted by the accumulated liver absorbed gamma dose, 10-year lag | |
|  | Liver absorbed alpha dose from internal exposure (Gy), 10-year lag | Person-years weighted by the accumulated liver absorbed alpha dose, 10-year lag | |
|  | Person-years | Person-years at risk | |
| Incident cases |  | 1: Liver MN  2: HCC  3: CC  4: AS  5: GBMN | |
